# *BRCA1* Alternative splicing landscape in breast tissue samples

**DOI:** 10.1186/s12885-015-1145-9

**Published:** 2015-04-03

**Authors:** Atocha Romero, Francisco García-García, Irene López-Perolio, Gorka Ruiz de Garibay, José A García-Sáenz, Pilar Garre, Patricia Ayllón, Esperanza Benito, Joaquín Dopazo, Eduardo Díaz-Rubio, Trinidad Caldés, Miguel de la Hoya

**Affiliations:** 1 Molecular Oncology Laboratoy, Instituto de Investigación Sanitaria San Carlos. Center affiliated to the Red Temática de Investigación Cooperativa (RD12/0036/006), Instituto Carlos III, Spanish Ministry of Economy and Competitivy, 28040 Madrid, Spain; 2 Medical Oncology Department, Hospital Puerta de Hierro, Madrid, Spain; 3 Computational Genomics Department, Centro de Investigación Príncipe Felipe, Valencia, Spain; 4 Breast Cancer and Systems Biology Unit, Translational Research Laboratory, Catalan Institute of Oncology (ICO), Bellvitge Institute for Biomedical Research (IDIBELL), L’Hospitalet del Llobregat, Instituto Carlos III, Spanish Ministry of Economy and Competitivy, Barcelona, 08908 Spain; 5 Medical Oncology Department. Hospital Clínico San Carlos. Department of Medicine. Faculty of Medicine, Universidad Complutense Madrid, Madrid, Spain; 6 Plastic Surgery Department, Hospital Clínico San Carlos, Madrid, Spain; 7 Functional Genomics Node, INB, CIPF, Valencia, Spain; 8 Centre for Biomedical Network Research on Rare Diseases (CIBERER), Valencia, Spain

**Keywords:** BRCA1, Splicing, Breast cancer

## Abstract

**Background:**

*BRCA1* is a key protein in cell network, involved in DNA repair pathways and cell cycle. Recently, the ENIGMA consortium has reported a high number of alternative splicing (AS) events at this locus in blood-derived samples. However, *BRCA1* splicing pattern in breast tissue samples is unknown. Here, we provide an accurate description of *BRCA1* splicing events distribution in breast tissue samples.

**Methods:**

*BRCA1* splicing events were scanned in 70 breast tumor samples, 4 breast samples from healthy individuals and in 72 blood-derived samples by capillary electrophoresis (capillary EP). Molecular subtype was identified in all tumor samples. Splicing events were considered *predominant* if their relative expression level was at least the 10% of the full-length reference signal.

**Results:**

54 *BRCA1* AS events were identified, 27 of them were annotated as *predominant* in at least one sample. Δ5q, Δ13, Δ9, Δ5 and ▼1aA were significantly more frequently annotated as predominant in breast tumor samples than in blood-derived samples. *Predominant* splicing events were, on average, more frequent in tumor samples than in normal breast tissue samples (P = 0.010). Similarly, *likely inactivating* splicing events (PTC-NMDs, Non-Coding, Δ5 and Δ18) were more frequently annotated as predominant in tumor than in normal breast samples (P = 0.020), whereas there were no significant differences for *other* splicing events (No-Fs) frequency distribution between tumor and normal breast samples (P = 0.689).

**Conclusions:**

Our results complement recent findings by the ENIGMA consortium, demonstrating that *BRCA1* AS, despite its tremendous complexity, is similar in breast and blood samples, with no evidences for tissue specific AS events. Further on, we conclude that somatic inactivation of *BRCA1* through spliciogenic mutations is, at best, a rare mechanism in breast carcinogenesis, albeit our data detects an excess of *likely inactivating* AS events in breast tumor samples.

**Electronic supplementary material:**

The online version of this article (doi:10.1186/s12885-015-1145-9) contains supplementary material, which is available to authorized users.

## Background

Germ-line inactivating mutations in the breast cancer susceptibility gene *BRCA1* (OMIM#113705) confer a marked hereditary predisposition to breast and ovarian cancer (HBOC). Yet, the role of *BRCA1* as a driving gene in sporadic breast cancer is, at best, far from clear. Early studies identified a very low proportion of somatically acquired *BRCA1* inactivating mutations in sporadic breast cancer [1]. Recent efforts aimed at elucidating the cancer genome have confirmed this view. According to the COSMIC database (http://cancer.sanger.ac.uk/cancergenome/projects/cosmic/; last accessed 08/01/2015), *BRCA1* somatic mutations are very rare, regardless of the breast cancer subtype analyzed (0.61% of breast cancer samples overall, 0.44% of basal-like breast cancers). While the analysis of somatic mutations does not support a major role for *BRCA1* in sporadic breast carcinogenesis, other evidences suggest that altered transcriptional regulation, rather than somatic mutations, might be involved in breast carcinogenesis [2-4]. For instance, it has been reported that sporadic basal-like tumors frequently down regulate *BRCA1* expression [2]. As far as we know the role of other possible *BRCA1* inactivating mechanisms (such as splicing alterations) in breast carcinogenesis has not been comprehensively explored.

Recently, the ENIGMA consortium has analyzed naturally occurring *BRCA1* alternative splicing (AS) in blood related RNA sources (commonly used for clinical splicing assays) [5], identifying up to 63 AS events, and supporting an AS model in which most non-mutually exclusive AS events are randomly combined into individual mRNAs molecules to produce probably hundreds of different RNA isoforms. To what extent these findings are tissue specific is not known. The ENIGMA study suggested that AS at the *BRCA1* locus is similar in blood and healthy breast tissue, albeit only one healthy breast tissue was available (one breast epithelia sample from a cosmetic surgery), thus limiting the power of the analysis [5].

In the present study, we have employed approaches previously developed by the ENIGMA consortia [5] to perform a comprehensive characterization of AS at the *BRCA1* locus in a cohort of 70 breast tumor samples from patients diagnosed as having locally advanced breast cancer, enrolled in a neoadjuvant clinical trial and whose tumors have been classified into intrinsic subtypes. For comparative purposes, we have characterized as well AS in 72 blood samples from healthy control individuals and 4 healthy breast tissues (cosmetic surgeries). As far as we know, this is the most comprehensive description of AS at the *BRCA1* locus reported so far in human breast cancer samples.

## Methods

### Study population

Tumor biopsy specimens were obtained from a set of 70 pre-treated patients, diagnosed as having locally advanced breast cancer. These patients participated in a neoadjuvant clinical trial (registered at the following Web site: http://www.clinicaltrials.gov; identifier NCT00123929) in which they were randomly assigned to receive four cycles of either doxorubicin (75 mg/m^2^body surface area) or docetaxel (Taxotere, Sanofi-Aventis, Spain) (100 mg/m^2^body surface area) every 3 weeks followed by surgery [6-9]. The clinical trial was approved by the Hospital Clínico San Carlos Ethics Committee, Madrid, Spain. Briefly, eligibility criteria included the following: women aged between 18 and 78 years; clinical stage IIB, IIIA, or IIIB breast cancer; and palpable breast tumors not amenable to breast-preserving surgery. Before the start of the trial, an informed consent was obtained from all participants. Clinico-pathological features of the study population are presented in Additional file 1: Table S1.

Additionally, breast tissue samples were obtained from four healthy individuals who underwent reduction mammoplasty. Similarly, whole blood was drawn from 72 healthy control donors (collected in EDTA tubes). All patients signed an informed consent for voluntary donation of biological samples for research to the Hospital Clínico San Carlos Biobank.

### Laboratory analysis

Tumor biopsy specimens were obtained before neoadjuvant chemotherapy. To check cellularity, an H&E image was obtained from all tumors; only samples with more than 80% tumor cells were used. Total RNA was extracted using the kit Qiagen RNeasy Mini Kit (Qiagen Inc., Valencia, CA), following the instructions of the manufacturer. Blood samples were kept ice-cold for a maximum of 30 minutes before RNA extraction. Total RNA was extracted from whole blood (200 μL) using a MagnaPure Compact workstation and MagnaPure RNA Isolation Kit according to the manufacturer’s protocol. The amount of RNA was assessed using a Nanodrop ND-1000 UV Spectrophotometer (Thermo Fisher Scientific, Wilmington, DE, USA). RNA integrity was assessed using a kit (RNA 6000 Nano Chip kit), followed by analysis with Bioanalyzer 2100 (Agilent Technologies, Santa Clara, CA). Total RNA, 200 ng, was used as a template to obtain first-strand cDNA using SuperScript First-Strand Synthesis System for RT-PCR (Invitrogen, Parsley, UK), following the manufacturer’s instructions.

**Figure 1:**
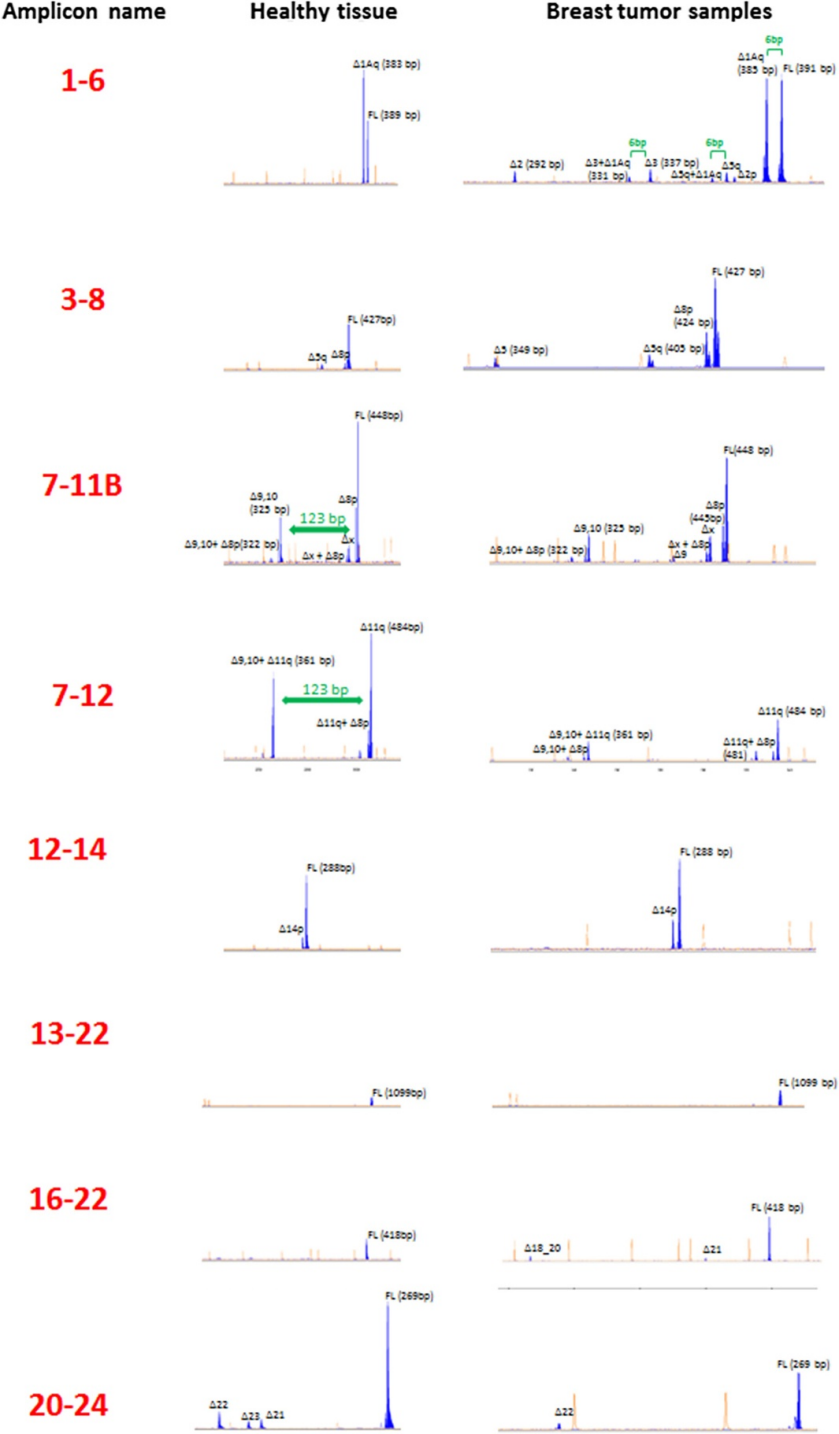
**Representative examples of each of the eight cDNA splicing assays.** Peak heights represent fluorescence intensity (scale on relative fluorescent units (RFU)). GeneScan 500-LIZ standard peaks are colored in orange. Peaks imputed to AS events are colored in blue. Capillary EP analysis permitted us to identify peaks imputed to transcripts carrying more than one independent splicing. For instance, assay 1–6 demonstrated that Δ1Aq (6 bp shorter than the FL) is combined with most of the splicing events visualized in this amplicon. Similarly, a peak of 322 bp in 7-11B assay demonstrated the presence of transcripts containing Δ8p and Δ9,10. As shown in 7-11B assay Δ9,10 is 123 bp shorter than the FL. In 7–12 assay a peak of 361 bp can be imputed to the combination of Δ9,10+ Δ11q since this peak is 123 bp shorter than Δ11q peak.

Comprehensive characterization of AS events at the *BRCA1* locus was performed by semi-quantitative capillary electrophoresis (capillary EP). The overall strategy has been reported elsewhere [5]. For the purpose of this study, we define AS events as those incorporating splice junctions not present in the reference transcript Ensemble ENST00000357654 (hereafter referred to as full-length transcript). In brief, eight combinations of forward and reverse primers located at exonic regions (hereafter referred to as splicing assays) were used to amplify cDNAs that later were analyzed by capillary EP. Figure 1 shows representative examples of each of the eight splicing assays. Primers sequences are shown in Additional file 1: Table S2. All assays were performed in a 25 ul reaction volume containing 1 ul of cDNA template, 2.5 ul of 10× PCR reaction buffer with 20 mM MgCl_2_ (Roche Diagnostics, Mannheim Germany), 0.2ul of FastStart Taq DNA polymerase (Roche Diagnostics, Mannheim Germany) 200 umol of each deoxynucleoside triphosphate and 0.25 umol of each primer. Thermal cycling consisted of an initial 10-minutes hold at 95°C, followed by 30-second hold at 95°C, 30-second hold at 58°C, and 60-second hold at 72°C for 33 cycles. In all cases, capillary EP analysis was performed in an ABI 3130 Genetic Analyzer (Applied Biosystems, Foster City, CA) using 50 cm capillary arrays filled with POP-7 and GeneScan 500-LIZ (Applied Biosystems, Foster City, CA) as internal size-standard. A standard electrophoresis protocol was used in all cases (temperature 60°C, injection 15 sec at 1.6KVolts, and running 1800 sec at 15KVolts). Peak heights under 100 RFU (Relative Fluorescent Units) were not considered for analyses. Size-calling and peak areas were analyzed with GeneMapper software version 4.0 (Applied Biosystems, Foster City, CA). Peak annotation was performed as previously reported [5].

Relative quantification of individual AS events (expressed as the average ratio between the peak area of that particular event and the peak area of the full-length signal in the corresponding assay) allowed us to classify AS events as *predominant* (≥10% of the full-length signal) or *minor* (<10%). AS events detected by the exons 7–12 assay were not classifiable because the full-length reference transcript containing exon 11 (>3300 bp) was not co-amplified. *BRCA1* exons were named according to the Breast Core Informative (BIC) database nomenclature [10], so that the 22 coding exons of the reference full-length transcript are numbered from 2 to 24 with no exon 4 defined. We have designated splicing events as previously reported [5,11,12] combining the following symbols: Δ (skipping), ▼ (retention), p (proximal), and q (distal). Structural and functional annotation of AS events was performed as previously described [5]. Briefly, functional annotation of *BRCA1* AS events includes: Non-Coding (splicing events eliminating the full-length start codon), PTC-NMDs (splicing events introducing Premature Termination Codons predicted to induce the Nonsense-Mediated RNA Decay pathway), No-FS (in-frame splicing events), FS-alternative STOP (frame-shift events generating PTCs not predicted to induce NMD as they are located in the most downstream *BRCA1* exons) and UTRs (splicing events modifying Un-Translated Regions). Further on, based on functional annotation, we classify AS events into *likely inactivating* and *other* AS events. The former category, referring to AS events that render the corresponding transcript unable to codify for functional *BRCA1* proteins, includes Non-Coding and PTC-NMD events, as well as two in-frame splicing events that target the critical RING (Δ5) and BRCT (Δ18) functional domains. By contrast, No-FS, FS-alternative STOP, and UTR AS events, with no obvious functional interpretation, were considered collectively as *other* AS events.

### IHC FISH, Tumor grading and subtype assignation

Paraffin-embedded tumor samples from core biopsy specimens were evaluated by immunohistochemical analysis for estrogen receptor (ER) (clone 1D5, 1:35; Dako Cytomation, Glostrup, Denmark) and progesterone receptor (PR) (clone PgR 636, 1:50; Dako Cytomation). After incubation with the primary antibodies, immunohistochemical studies were performed using the Autostainer link 48 (Dako Cytomation, Carpinteria, CA). The cut points for ER and PR positivity were established at 1% or greater of stained cells. Slides of all tumors were reviewed for diagnostic reassessment of the tumor histotype and histological grade, in a blinded fashion. The amplification of *ERBB2* was measured by FISH (fluorescence in situ hybridization).The probes used were as follows: Centromere enumeration probe 17, labeled in green; and locus-specific identifier *ERBB2* probe, labeled in orange (Vysis-Abbott, Downers Grove, IL). Slides were prepared according to the manufacturer’s instructions. A positive result was defined as an *ERBB2* gene/chromosome 17 ratio of 2.2 or greater. A minimum of 100 nuclei were counted per case.

Gene expression data from previously hybridized gene expression microarrays [6,13] (available in the Gene Expression Omnibus repository database under accession number GSE21997) was used for intrinsic subtype assignment. Breast cancer molecular subtypes were identified using the PAM50 and the Claudin-low (CLP) subtype predictors as previously described [14,15].

### Statistical analysis

All analysis where restricted to the subgroup of predominant splicing events (events representing >10% of the full-length signal in at least one tumor sample) exclusively. Association between two categorical variables was assessed using the *Χ*^2^ or Fisher exact test in cases in which more than 25% of the expected values were less than five. P-values were corrected for multiple comparisons using False Discovery Rate (FDR) method [16]. For quantitative variables, comparisons were assessed by T-student test or Mann Whitney test when appropriate. P < 0.05 was considered for statistical significance. The statistical analysis was performed using software R 3.0.1.

## Results

**Figure 2:**
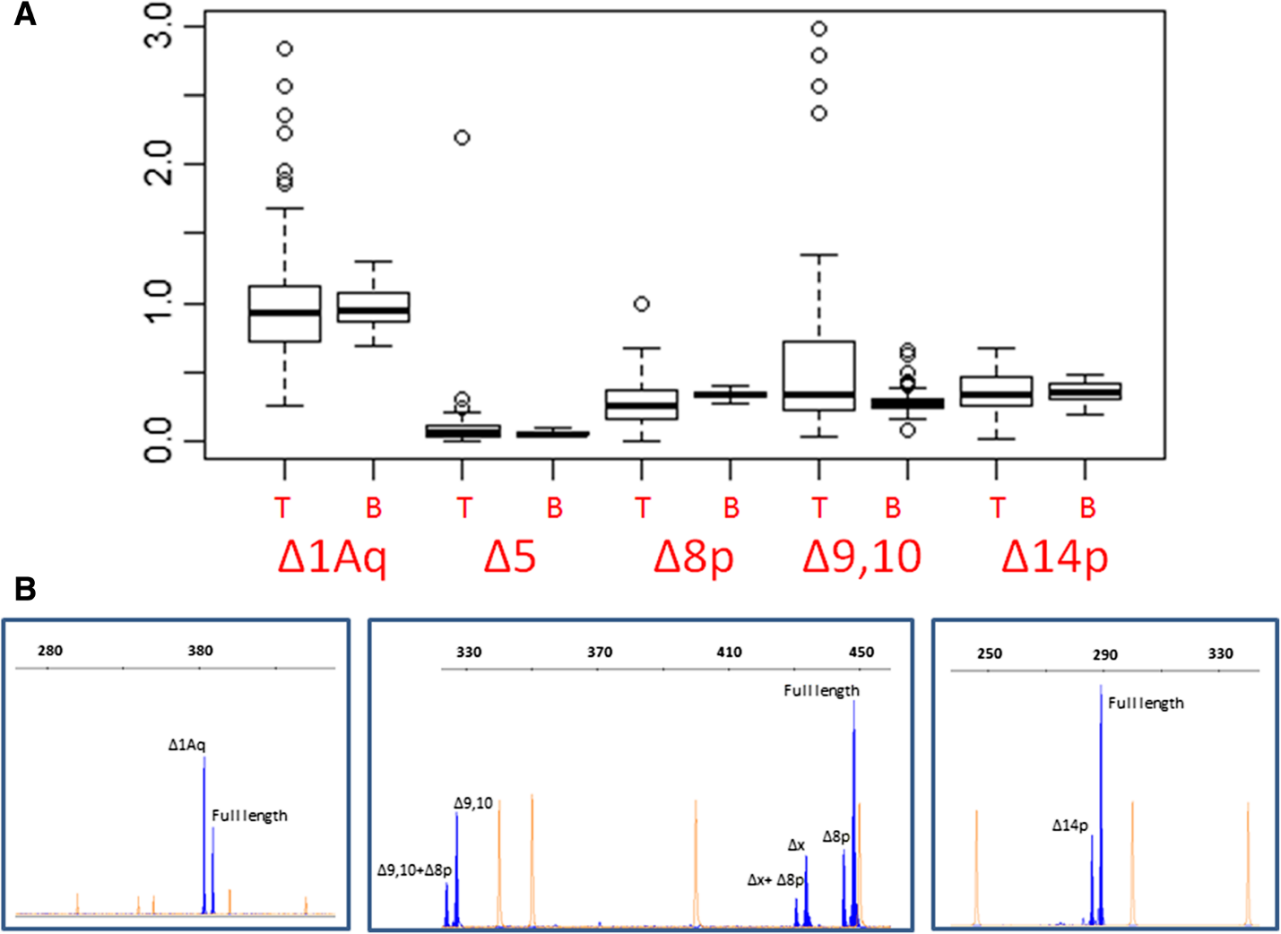
**Relative quantification of predominant splicing events. (A)**. The chart shows the ratios between peak areas of alternative splicing events and peak areas of the reference full-length transcripts in tumor samples (T) and blood-derived samples **(B)**. *(P < 0.01) **(B)**. Representative example of a cDNA splicing assays showing Δ1Aq, Δ8p, Δ9,10 and, Δ14p peak areas (blue). The assay allowed us to identify transcripts with different combinations of splicing events such as Δ9, 10 + Δ8p. Interestingly, the assay predicts the existence of another splicing event involving ±16 bp (X in the figure).

Overall, we have identified in breast tissue samples most of the *BRCA1* AS events previously identified by the ENIGMA consortium in blood-derived samples (54 out of 63, see Additional file 1: Table S3) [5]. As previously observed [5], most AS events were rather minor in most analyzed samples, with the notable exception that Δ1Aq, Δ8p, Δ9,10, Δ14p and Δ5q (Table 1) were frequently identified as *predominant* AS events, regardless of the tissue of origin. Further on, the relative quantification of these AS events is similar in breast tumor and blood samples, with Δ1Aq expressed on average at similar levels than the full-length reference and Δ8p, Δ9,10 and Δ14p, signals representing roughly 35%. Nonetheless, Δ5q levels were higher in breast tumor samples (P < 0.01) (Figure 2). Equally relevant, we have not identified any breast specific AS event, neither in healthy nor in malignant samples. Taken together, the data suggests that, AS events at the *BRCA1* locus in human breast tissue and blood are similar, with no evidence for tissue specific AS events. These observations support the clinical use of blood samples as an adequate surrogate for testing the spliciogenic effect of *BRCA1* genetic variants. Further on, our data suggests as well that somatic inactivation of *BRCA1* through the acquisition of spliciogenic mutations is, at best, an uncommon mechanism in breast carcinogenesis.

**Table 1:** **Predominant splicing events distribution by tissue**

				*Breast tumor*	*Healthy breast tissue*	*Blood-derived samples*		
*Variant name*	*HGVS description*	*Functional annotation*	*Biotype*	*Frequency predominant*	*Frequency predominant*	*P* ^*1*^	*Frequency predominant*	*P* ^*2*^
**Δ8p**	c.442_444del3	No FS	Splice acceptor shifts	1.000	1.000	NS	1.000	NS
**Δ14p**	c.4358_4360del3	No FS	Splice acceptor shifts	1.000	1.000	NS	1.000	NS
**Δ1Aq**	c.-25_-20del6	UTR	Splice donor shifts	1.000	1.000	NS	1.000	NS
**Δ9,10**	c.548_670del123	No FS	multi-cassette	0.971	1.000	NS	0.988	NS
**Δ5q**	c.191_212del22	PTC-NMD	Splice donor shifts	0.857	0.750	NS	0.391	0.001
**Δ13**	c.4186_4357del172	PTC-NMD	Cassette	0.357	0.000	NS	0.000	0.049
**Δ9**	c.548_593del46	PTC-NMD	Cassette	0.203	0.000	NS	0.000	0.0175
**Δ5**	c.135 _212del78	No FS	Cassette	0.200	0.250	NS	0.000	0.0001
**Δ2**	c.-19_80del99	Non-Coding	Cassette	0.200	0.250	NS	0.333	NS
**▼1aA**	c.-20 + 1_-20 + 89ins89	UTR	Splice donor shifts	0.157	0.000	NS	0.000	0.006
**Δ22**	c.5333_5406del74	FS-alternative STOP	Cassette	0.129	0.000	NS	0.031	NS
**Δ2p**	c.-19_-7del13	UTR	Splice acceptor shifts	0.114	0.000	NS	0.059	NS
**Δ10**	c.594_670del77	PTC-NMD	Cassette	0.087	0.000	NS	0.000	0.07
**▼4**	c.135-4047_135-3932ins116	PTC-NMD	Cassette	0.086	0.000	NS	0.000	0.07
**Δ8_10**	c.442_670del229	PTC-NMD	multi-cassette	0.072	0.000	NS	0.000	0.082
**Δ3**	c.81_134del54	PTC-NMD	Cassette	0.071	0.000	NS	0.071	NS
**Δ21**	c.5278_5332del55	PTC-NMD	Cassette	0.071	0.000	NS	0.000	NS
**Δ15**	c.4485_4675del190	PTC-NMD	Cassette	0.071	0.000	NS	0.000	NS
**Δ21_23**	c.5278_5467de	FS-alternative STOP	multi-cassette	0.071	0.000	NS	0.034	NS
**Δ2,3**	c.-19_134del153	Non-Coding	multi-cassette	0.043	0.000	NS	0.023	NS
**▼13A**	c.4358-2785_4358-2729ins66	No FS	Cassette	0.043	0.000	NS	0.000	NS
**Δ21,22**	c.5278_5406del129	No FS	multi-cassette	0.043	0.000	NS	0.000	NS
**Δ8,9**	c.442_593del152	PTC-NMD	multi-cassette	0.014	0.000	NS	0.000	NS
**Δ17**	c.4987_5074del88	PTC-NMD	Cassette	0.014	0.000	NS	0.000	NS
**Δ22,23**	c.5333_5467del135	FS-alternative STOP	multi-cassette	0.014	0.000	NS	0.000	NS
**Δ13p**	c.4186_4188del3	No FS	Splice acceptor shifts	0.014	0.000	NS	0.000	NS
**Δ15_17**	c.4485_5074del590	PTC-NMD	multi-cassette	0.014	0.000	NS	0.000	NS

P^1^ for significance of Fisher exact test for differences between observed and expected values of AS events frequency distribution in breast tumor samples and healthy breast samples. P^2^ for significance of ×^2^ statistic or Fisher exact test, when appropriate, for differences between observed and expected values of AS events frequency distribution in breast tumor samples and blood-derived samples. Note that none of the 27 AS events listed is annotated more frequently as predominant in blood- derived sample.

While the above data indicates that, on average, AS at the *BRCA1* locus is similar in breast and blood samples, Δ5q findings prompted us to explore the possibility that more subtle splicing alterations targeting individual AS events in individual breast tumor samples do exist. To begin with, we have identified up to 27 AS events that were annotated as *predominant* (>10% of the reference full-length signal) in at least one tumor sample (Table 1). Focusing on individual AS events, we found that seven (Δ5q, Δ13, Δ9, Δ5, ▼1aA, Δ10 and ▼4, all of them *likely inactivating*) were more frequently annotated as predominant in breast tumor samples than in blood-derived samples, albeit in the case of Δ10 and ▼4 the findings did not reach statistical significance (Table 1). After FDR correction, the differences in Δ5q and Δ5 frequency distribution between blood and tumor samples remained significant (P = 0.014 and P = 0.003 respectively).

Similarly, we observed that, on average, the absolute number of predominant splicing events per sample was higher in tumor samples than in healthy breast tissue samples (6.8 vs. 5.0; P = 0.010). The same was true for peaks corresponding to the combination of two or more splicing events (8.9 vs. 5.7; P = 0.004). Additionally, we found that *likely inactivating* AS events were, on average, more frequently annotated as predominant in breast tumor samples than in healthy breast samples (2.6 vs 1.0; P = 0.020), while the same effect was not true for *other* AS events (4.1 vs 4.0; P = 0.689). Some representative examples of *likely inactivating* AS events occurring in breast tumor samples are displayed in Additional file 1: Figure S1. Overall, the data suggests an excess of *likely inactivating* AS events in breast cancer cells.

Finally, we explore the possibility that certain AS events annotated as predominant in individual tumor samples were associated with the intrinsic subtype, as defined by PAM50 + CLP genomic profiling, but we did not identify any obvious association.

## Discussion

To our knowledge, this is the most comprehensive description of AS at the *BRCA1* locus reported so far in human breast samples. To perform this analysis, we took advantage of recent studies conducted by the ENIGMA consortium for the characterization of naturally occurring AS at the *BRCA1* locus in blood related samples [5]. According to the ENIGMA consortium, pervasive AS at the *BRCA1* locus is observed in blood related samples, with up to 63 individual AS events identified [5]. This observation was consistent with several genome-wide analyses that have identify a strong association of high level AS loci with intrinsically disordered protein/domains (IDPs/IDDs), IDPs/IDDs with Hub proteins, and Hub proteins with disease [17-19].

In the present study, we analyze to what extent this high level of AS is observed also in breast related samples. With this aim, we have characterized *BRCA1* AS in 70 breast tumor samples and 4 healthy breast tissue samples. For ethical reasons, it is difficult to access healthy human breast samples, so that we have performed the study in a very small cohort. Overall, our study concludes that AS at the *BRCA1* locus is similar in breast and blood samples. First, most AS events previously identified in blood, 54 out of 63, have been identified as well in healthy and tumor breast samples. Second, we have not identified any novel AS event not previously described in blood. Third, up to four AS events (Δ1Aq, Δ8p, Δ9,10, Δ14p) emerge as *predominant* in almost all samples of both tissues. Four, the relative semi-quantification of these *predominant* AS events reveals similar profiles in both tissues, with Δ1Aq representing roughly 90% of the reference full-length signal, Δ8p, Δ9,10, and Δ14p representing 30% (Figure 2). As a corollary, we conclude that somatic inactivation of *BRCA1* through spliciogenic mutations is, at best, a rare mechanism in breast carcinogenesis.

We have concluded that, on average, AS at the *BRCA1* locus in breast and blood samples is very similar. Yet, in the present study we addressed as well the possibility that subtle quantitative alterations involving certain AS events were detectable in individual breast tumor samples. Due to the complexity of such analysis, in the present study we have restricted ourselves to compare the average number of *predominant* AS events detected in individual breast and blood samples. Interestingly, our data supports an excess of *predominant* AS events in tumor samples, in particular AS events annotated as *likely inactivating*. Since we have analyzed very few healthy breast samples, we cannot rule out the possibility that this finding (and excess of *likely inactivating* AS events in breast tumor samples) reflects tissue specific AS regulation, rather than a tumor specific phenotype acquired in the carcinogenic process. Further on, according to our data *likely inactivating* splicing events are not associated with any particular breast cancer subtype. Splicing deregulation represents a relevant ethiopathogenic mechanism of some diseases, including cancer [20]. Yet, further studies are needed to establish a role, if any, for *BRCA1 likely inactivating* AS events in sporadic breast carcinogenesis.

A catalogue of AS events occurring at the *BRCA1* locus in breast tissue sample is an essential requirement for the design of adequate RNA splicing assays assessing the pathogenicity of *BRCA1* sequence variants. Indeed, our findings of similar AS events in breast and blood samples somehow validate the use of blood-derived samples for *in vitro* splicing assays testing the clinical relevance of *BRCA1* sequence variants, a key issue in molecular diagnosis [12]. In addition, the results presented here will help researchers to analyze and validate data from RNAseq experiments that are becoming increasingly widespread. Moreover, tools for functional annotations of novel sequences (novel transcripts) and the analyses of annotation data are clearly warranted. In this way, our data might be useful for the development of such tools.

## Conclusions

Our results complement recent findings by the ENIGMA consortium, demonstrating that *BRCA1* AS, despite its tremendous complexity, is very similar in breast and blood samples, with no evidences for tissue specific AS events. Further on, we conclude that somatic inactivation of *BRCA1* through spliciogenic mutations is, at best, a rare mechanism in breast carcinogenesis, albeit our data detects an excess of *likely inactivating* AS events in breast tumor samples.

## Additional file

Additional file 1: Table S1. Clinicopathological characteristics of the 70-breast tumor sample cohort. **Table S2.** Primer sequences and full length fragment size. **Table S3.** Splicing events previously identified in blood-derived samples. The 2^nd^ column indicates whether the splicing event have been cloned and sequenced or directly sequenced from the splicing assay (yes) or imputed to predicted size-fragments according to Ensemble reference transcript ENST00000357654 (no). For further details see reference [5]. The 3^rd^ column shows the AS events that have been detected in at least one sample of the present study. Not analyzed refers to AS events that couldn’t be identified by the analytical approach carried out. **Figure S1.** PTC-NMD predominant splicing events in breast tumor samples.

## Abbreviations

(AS): Alternative splicing
(Capillary EP): Capillary electrophoresis
(ER): Estrogen receptor
(HBOC): Hereditary Breast and Ovarian Cancer
(No-FS): in-Frame splicing events
(PR): Progesterone receptor
(PTC-NMD): Splicing events introducing premature termination codons predicted to induce the nonsense-mediated
RNA: Decay pathway

## References

[1] van der Looij M, Cleton-Jansen AM, van Eijk R, Morreau H, van Vliet M, Kuipers-Dijkshoorn N (2000). A sporadic breast tumor with a somatically acquired complex genomic rearrangement in *BRCA1*. Genes Chromosomes Cancer.

[2] Turner NC, Reis-Filho JS, Russell AM, Springall RJ, Ryder K, Steele D (2007). BRCA1 dysfunction in sporadic basal-like breast cancer. Oncogene.

[3] Wilson CA, Ramos L, Villaseñor MR, Anders KH, Press MF, Clarke K (1999). Localization of human BRCA1 and its loss in high-grade, non-inherited breast carcinomas. Nat Genet.

[4] Di LJ, Fernandez AG, De Siervi A, Longo DL, Gardner K (2010). Transcriptional regulation of BRCA1 expression by a metabolic switch. Nat Struct Mol Biol.

[5] Colombo M, Blok MJ, Whiley P, Santamariña M, Gutiérrez-Enríquez S, Romero A, Garre P, Becker A, Smith LD, De Vecchi G, Brandão RD, Tserpelis D, Brown M, Blanco A, Bonache S, Menéndez M, Houdayer C, Foglia C, Fackenthal JD, Baralle D, Wappenschmidt B; kConFaB Investigators, Díaz-Rubio E, Caldés T, Walker L, Díez O, Vega A, Spurdle AB, Radice P, De La Hoya M. Comprehensive annotation of splice junctions supports pervasive alternative splicing at the BRCA1 locus: a report from the ENIGMA consortium. Hum Mol Genet. 2014;15:23:3666–80. 10.1093/hmg/ddu075 24569164

[6] Martin M, Romero A, Cheang MC, López García-Asenjo JA, García-Saenz JA, Oliva B (2011). Genomic predictors of response to doxorubicin versus docetaxel in primary breast cancer. Breast Cancer Res Treat.

[7] Romero A, Martín M, Cheang MC, López García-Asenjo JA, Oliva B, He X (2011). Assessment of topoisomerase IIa status in breast cancer by quantitative PCR, gene expression microarrays, immunohistochemistry, and fluorescence in situ hybridization. Am J Pathol.

[8] Romero A, Martín M, Oliva B, de la Torre J, Furio V, de la Hoya M (2012). Glutathione S Transferase P1 c.313A > G polymorphism could be useful in the prediction of doxorubicin response in breast cancer patients. Ann Oncol.

[9] Romero A, García-Sáenz JA, Fuentes-Ferrer M, López Garcia-Asenjo JA, Furió V, Román JM (2013). Correlation between response to neoadjuvant chemotherapy and survival in locally advanced breast cancer patients. Ann Oncol.

[10] Szabo C, Masiello A, Ryan JF, Brody LC (2000). The breast cancer information core: database design, structure, and scope. Hum Mutat.

[11] de Garibay GR, Acedo A, García-Casado Z, Gutiérrez-Enríquez S, Tosar A, Romero A (2014). Capillary electrophoresis analysis of conventional splicing assays: IARC analytical and clinical classification of 31 BRCA2 genetic variants. Hum Mutat.

[12] Whiley PJ, de la Hoya M, Thomassen M, Becker A, Brandão R, Pedersen IS (2014). Comparison of mRNA splicing assay protocols across multiple laboratories: recommendations for best practice in standardized clinical testing. Clin Chem.

[13] Romero A, Prat A, García-Sáenz JA, Del Prado N, Pelayo A, Furió V (2014). Assignment of tumor subtype by genomic testing and pathologic-based approximations: implications on patient's management and therapy selection. Clin Transl Oncol.

[14] Parker JS, Mullins M, Cheang MC, Leung S, Voduc D, Vickery T (2009). Supervised risk predictor of breast cancer based on intrinsic subtypes. J Clin Oncol.

[15] Prat A, Parker JS, Karginova O, Fan C, Livasy C, Herschkowitz JI (2010). Phenotypic and molecular characterization of the claudin-low intrinsic subtype of breast cancer. Breast Cancer Res.

[16] Benjamini Y, Hochberg Y (1995). Controlling the false discovery rate: a practical and powerful approach to multiple testing. J R Stat Soc Ser B.

[17] Cortese MS, Uversky VN, Dunker AK (2008). Intrinsic disorder in scaffold proteins: getting more from less. Prog Biophys Mol Biol.

[18] Uversky VN, Oldfield CJ, Midic U, Xie H, Xue B, Vucetic S, Iakoucheva LM, Obradovic Z, Dunker AK. Unfoldomics of human diseases: linking protein intrinsic disorder with diseases. BMC Genomics. 2009;10:Suppl 1-S7. 10.1186/1471-2164-10-S1-S7 PMC2709268 19594884

[19] Kornblihtt AR, Schor IE, Alló M, Dujardin G, Petrillo E, Muñoz MJ (2013). Alternative splicing: a pivotal step between eukaryotic transcription and translation. Nat Rev Mol Cell Biol.

[20] Biamonti G, Catillo M, Pignataro D, Montecucco A, Ghigna C (2014). The alternative splicing side of cancer. Semin Cell Dev Biol.

