# Supplementary material for: BRCA1 Alternative splicing landscape in breast tissue samples
Source: BMC Cancer. 2015 Apr 3;15:219. doi: 10.1186/s12885-015-1145-9 (PMC4393587; doi:10.1186/s12885-015-1145-9)
Supplement: Additional file 1: Table S1. — Clinicopathological characteristics of the 70-breast tumor sample cohort. Table S2. Primer sequences and full length fragment size. Table S3. Splicing events previously identified in blood-derived samples. The 2nd column indicates whether the splicing event have been cloned and sequenced or directly sequenced from the splicing assay (yes) or imputed to predicted size-fragments according to Ensemble reference transcript ENST00000357654 (no). For further details see reference [5]. The 3rd column shows the AS events that have been detected in at least one sample of the present study. Not analyzed refers to AS events that couldn’t be identified by the analytical approach carried out. Figure S1. PTC-NMD predominant splicing events in breast tumor samples. [file 12885_2015_1145_MOESM1_ESM.docx]

| ***Clinicopathologic Characteristics*** |  |
| --- | --- |
|  |  |
| *Age at diagnosis (years)* |  |
| *Mean (range)* | 53.4 (27-77) |
| *Pre-treatment tumor size (cm)* |  |
| *Median (range)* | 6 (2-12) |
| *Histology (n (%))* |  |
| *Ductal* | 61 (87%) |
| *Lobular* | 7 (10%) |
| *Other* | 2 (3%) |
| *Histologycal grade (n (%))* |  |
| *I and II* | 42 (60%) |
| *III* | 28 (40%) |
| *UICC stage (n (%))* |  |
| *II* | 23 (33%) |
| *IIIA* | 19 (27%) |
| *IIIB* | 28 (40%) |
| *Lymph nodes status at diagnosis (n (%))* |  |
| *positive* | 44 (63%) |
| *negative* | 26 (37%) |
| *Lymphocite infiltration (n (%))* |  |
| *positive* | 19 (27%) |
| *negative* | 51 (73%) |
| *Subtype distribution (PAM50+CL) (n(%))* |  |
| *Luminal A* | 14 (20%) |
| *Luminal B* | 21 (30%) |
| *Basal-like* | 10 (14%) |
| *Her2-enrriched* | 9 (13%) |
| *Caludin Low* | 11 (16%) |
| *Normal-like* | 5 (7%) |
| *Neoadjuvant treatment branch (n (%))* |  |
| *docetaxel* | 29 (41%) |
| *doxorubicin* | 41 (59%) |
| *Neoadyuvant outcome (RCB class) (n (%))* |  |
| *RCB-III* | 28 (40%) |
| *RCB-II* | 28 (40%) |
| *RCB-I* | 4 (6%) |
| *RCB-0* | 10 (14%) |

Table S1. Clinicopathological characteristics of the 70-breast tumor sample cohort

| Amplicon name | Foward primer | Reverse primer | FL size |
| --- | --- | --- | --- |
| 1A-6 | GACAGGCTGTGGGGTTTCT | TCCAAACCTGTGTCAAGCTG | 391 |
| 3-8 | TCAAGGAACCTCTCTCCACA | GGTTGTATCCGCTGCTTTGT | 427 |
| 7-11B | CATCCAAAGTATGGGCTACAGA | TGGCTCCACATGCAAGTTTG | 448 |
| 7-12 | CATCCAAAGTATGGGCTACAGA | CTGAGAGGATAGCCCTGA | 3793 |
| 12-14 | GCGTCTCTGAAGACTGCTCA | AAAGGCCTTCTGGATTCTGG | 288 |
| 13-22 | ATGGGAGCCAGCCTTCTAAC | CACAGCTGTACCATCCAT | 1099 |
| 16-22 | AAAGAATGTCCATGGTGGTG | CACAGCTGTACCATCCAT | 418 |
| 20-24 | AGAAACCACCAAGGTCCAAAG | ACCACAGGTGCCTCACAC | 269 |

Table S2. Primer sequences and full length fragment size

Table S3. Splicing events previously identified in ***blood-derived*** samples. The 2^nd^ column indicates whether the splicing event have been cloned and sequenced or directly sequenced from the splicing assay (yes) or imputed to predicted size-fragments according to Ensemble reference transcript ENST00000357654 (no). For further details see reference **[5]**. The 3^rd^ column shows the AS events that have been detected in at least one sample of the present study. Not analyzed refers to AS events that couldn’t be identified by the analytical approach carried out.


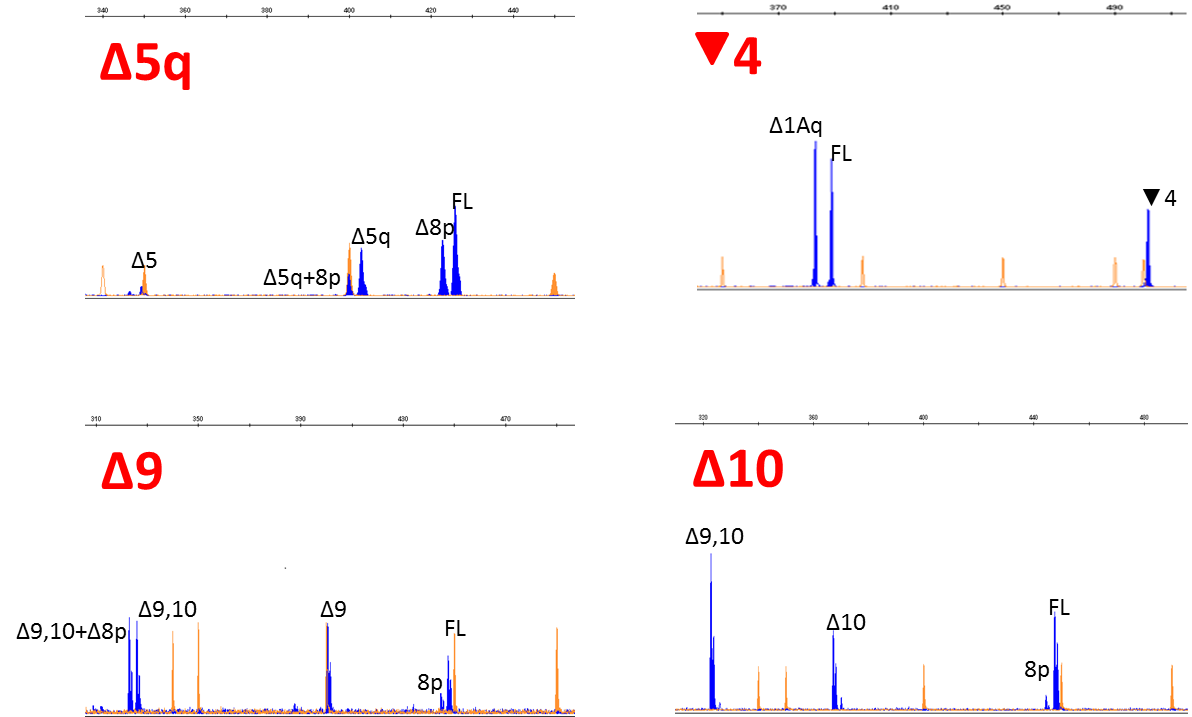


Supplemental Figure 1. PTC-NMD predominant splicing events in breast tumor samples.
